# Conscious Movement Processing, Fall-Related Anxiety, and the Visuomotor Control of Locomotion in Older Adults

**DOI:** 10.1093/geronb/gbaa081

**Published:** 2020-08-06

**Authors:** Toby J Ellmers, Adam J Cocks, Elmar C Kal, William R Young

**Affiliations:** 1 College of Health and Life Sciences, Brunel University London, UK; 2 Centre for Cognitive Neuroscience, Brunel University London, UK; 3 School of Sport and Health Sciences, University of Exeter, UK

**Keywords:** Eye tracking, Fear of falling, Gait, Internal focus, Visual search

## Abstract

**Objectives:**

Older adults anxious about falling will often consciously process walking movements in an attempt to avoid falling. They also fixate their gaze on the present step rather than looking ahead to plan future actions. The present work examined whether conscious movement strategies result in such restricted visual planning.

**Methods:**

A total of 18 community-dwelling older adults (age^mean^ = 71.22; *SD* = 5.75) walked along a path and stepped into two raised targets. Repeated-measures analyses of variance were used to compare gaze behavior and movement kinematics when participants walked: (a) at baseline (ground level); (b) under conditions designed to induce fall-related anxiety (walkway elevated 0.6 m); and (c) in the absence of anxiety (ground level), but with explicit instructions to consciously process movements.

**Results:**

Participants reported increased conscious movement processing when walking both on the elevated walkway (fall-related anxiety condition) and at ground level when instructed to consciously process gait. During both conditions, participants altered their gaze behavior, visually prioritizing the immediate walkway 1–2 steps ahead (areas needed for the on-line visual control of individual steps) at the expense of previewing distal areas of the walking path required to plan future steps. These alterations were accompanied by significantly slower gait and increased stance durations prior to target steps.

**Conclusions:**

Consciously processing movement (in the relative absence of anxiety) resulted in gaze behavior comparable to that observed during conditions of fall-related anxiety. As anxious participants also self-reported directing greater attention toward movement, this suggests that fall-related anxiety may disrupt the visual control of gait through increased conscious movement processing.

Given both the high prevalence of falls in older adults (Lord, Ward, Williams, & Anstey, 1993; Tromp et al., 2001), and the negative psychological, physical, and social impact associated with experiencing a fall (Białoszewski et al., 2008; Hadjistavropoulos, Delbaere, & Fitzgerald, 2011), it is not surprising that older adults will often consciously process walking movements in an attempt to avoid falling (Wong, Masters, Maxwell, & Abernethy, 2008). However, while some degree of conscious, controlled processing is required for older adults to maintain balance (Boisgontier et al., 2013)—particularly during challenging or complex tasks, or in individuals for whom “automatic” control processes are defective—excessive conscious processing may ironically compromise the control of balance and gait. For example, Uiga et al. (2020) described that older adults who consciously process their movements will take longer to plan and prepare stepping movements, yet display increased stepping errors likely to reduce safety. Relatedly, Mak, Young, Chan, and Wong (2020) reported both increased gait variability and reduced postural stability in older adults during experimental conditions of conscious movement processing. There is also considerable evidence that conscious processing will disrupt postural stability during static balance tasks (Polskaia, Richer, Dionne, & Lajoie, 2015; Richer, Saunders, Polskaia, & Lajoie, 2017; Wulf, 2013). These effects are the likely consequence of such conscious strategies disrupting the subconscious (“automatic”) lower level processes through which complex, highly coordinated motor actions—such as balance and locomotion—are typically regulated (Clark, 2015; Masters & Maxwell, 2008; Wulf, 2013).

Conscious movement strategies may also reduce safety during locomotion through mechanisms other than direct disruption to movement execution. For example, research indicates that directing attention internally toward movement reduces the walker’s ability to attend to their environment (Uiga, Capio, Wong, Wilson, & Masters, 2015; Young, Olonilua, Masters, Dimitriadis, & Williams, 2016). More specifically, Clark (2015) suggests that during “. . . the [conscious] control of the basic walking pattern, there is a heightened risk that hazards may be overlooked or ignored [. . .] resulting in slips, trips, collisions and falls” (p. 2). Indeed, recent research highlights that young adults who consciously process their walking movements will do so at the expense of visually fixating upcoming stepping constraints (Ellmers & Young, 2019).

These findings imply that conscious movement strategies may reduce the walker’s ability to utilize vision in a feedforward manner (ie, looking multiple steps ahead to facilitate the planning of future stepping movements); perhaps due to a prioritization of on-line visual control (Ellmers & Young, 2019). On-line control—the use of vision to regulate an ongoing action—is important for fine-tuning stepping movements (Chapman & Hollands, 2006a, 2007; Reynolds & Day 2005). However, the ability to preplan movements using feedforward visual control is critically important for maximizing gait stability, safety, and efficiency (Barton, Matthis, & Fajen 2017; Matthis, Barton, & Fajen 2015, 2017; Matthis & Fajen 2014). Thus, by reducing an individual’s opportunity to use feedforward control, conscious movement processing may reduce walking safety in older adults. We have recently reported indirect evidence for this hypothesis, by showing that reduced feedforward planning is associated with both heightened conscious movement processing, and increased stepping errors, in older adults at a high risk of falling (Ellmers, Cocks, & Young, 2020). Yet, the direct (ie, causal) influence of conscious movement processing on older adults visuomotor control of locomotion remains unknown.

A common trigger for conscious movement processing in older adults is fall-related anxiety (Ellmers et al., 2019; Ellmers, Cocks, & Young, 2020; Young & Williams 2015). Older adults who are anxious will often also display disrupted visual search during adaptive locomotion (Ellmers et al., 2020; Young, Wing, & Hollands, 2012; Young & Williams, 2015). For example, much like young adults during experimentally induced conditions of conscious movement processing (Ellmers & Young, 2019), older adults who are anxious will display restricted visual planning whereby they fixate immediate stepping constraints at the expense of previewing future stepping actions. It is, however, unclear whether conscious movement strategies result in these restricted patterns of visual search. For example, fall-related anxiety may also alter visual search behaviors via processes other than conscious movement processing, such as a gaze bias for threatening stimuli (Staab, 2014) or changes in sensory processing associated with increased arousal (Adkin & Carpenter, 2018).

Thus, the present study sought to (a) confirm that previously observed anxiety-related changes in visual search behavior occur in conjunction with increased self-reported conscious movement processing and (b) manipulate conscious movement processing (independent from fall-related anxiety) to test for a causal link between this movement strategy and altered visual search. Healthy older adults completed an adaptive locomotion task which required them to step into two raised targets. Walks were completed during conditions designed to induce (a) fall-related anxiety (postural threat) and (b) conscious movement processing, independent from threat-related anxiety. Walks were also completed under baseline conditions. Following our assertion that anxiety-related changes in visual search are, at least in part, driven by conscious movement processing, we predicted that visual search behaviors would be comparable during conditions of postural threat and conscious movement processing. Specifically, we predicted that participants would direct preferential attention toward immediate areas of the walkway needed to consciously process discrete stepping movements, at the expense of previewing future stepping constraints. This prediction does not deny the likely influence of countless factors associated with increased physiological arousal (eg, altered sensory integration) that could also contribute to changes in visual search behavior, but merely seeks to evaluate whether a causal link exists between conscious movement processing and altered visual search during locomotion.

## Methods

### Participants

A total of 18 community-dwelling older adults without a recent history of falling (in the previous 12 months) were recruited from the “low fall-risk” subset of a previous study (Ellmers et al., 2020).^1^ Previous research exploring the effect of experimentally induced conscious movement processing on visual search behavior during locomotion has reported large effect sizes (*r* = *Z*/√*N*) for key, comparable variables (Ellmers & Young, 2019). Consequently, a power analysis determined that 14 participants would be required to obtain 80% power for a repeated-measures analysis of variance (ANOVA). Institutional ethical approval was obtained from the local ethics committee and the research was carried out in accordance with the principles laid down by the Declaration of Helsinki. All participants provided written informed consent prior to participation. The study was not preregistered.

All participants were free from any neurological, cardiovascular, or musculoskeletal impairment that prohibited them from walking 10 m without a walking aid. Participants were excluded if they demonstrated major cognitive impairment (MiniCog score of <3; Borson, Scanlan, Brush, Vitaliano, & Dokmak, 2000; Borson, Scanlan, Chen, & Ganguli, 2003; Borson, Scanlan, Watanabe, Tu, & Lessig, 2006), or if they were currently prescribed anxiety or dizziness medication. Exclusion criteria included: static visual acuity of less than 20/40, significant deficits in contrast sensitivity (log contrast sensitivity score of 1) or any other reported ocular condition, other than mild cataracts. Individuals who required the use of corrected lenses during daily locomotion were prescreened for compatibility with the eye-tracking equipment, and invited to participate if it was possible to calibrate the eye-tracker over their glasses (as per Ellmers et al., 2020). Two participants completed walks while wearing habitually corrected single-distance lenses,^2^ whereas the remainder completed the protocol without correcting lenses. Prior to the experimental protocol, participants also completed standard clinical assessments of both functional balance/mobility (Timed up and Go; Podsiadlo & Richardson, 1991) and concerns about falling (Falls Efficacy Scale-International; Yardley et al., 2005). Participant demographics are reported in Table 1.

**Table 1.** Participant Characteristics

Measure	Mean (± *SD*)
Age	71.22 (± 5.75)
Gender (males)	7/18
Number of fallers (past 12 months)	0
Timed up and go (s)	9.38 (± 1.28)
MiniCog (0–5)	4.39 (± 0.78)
Falls Efficacy Scale-International (16–64)	18.83 (± 2.20)

### Protocol

**Figure 1.**
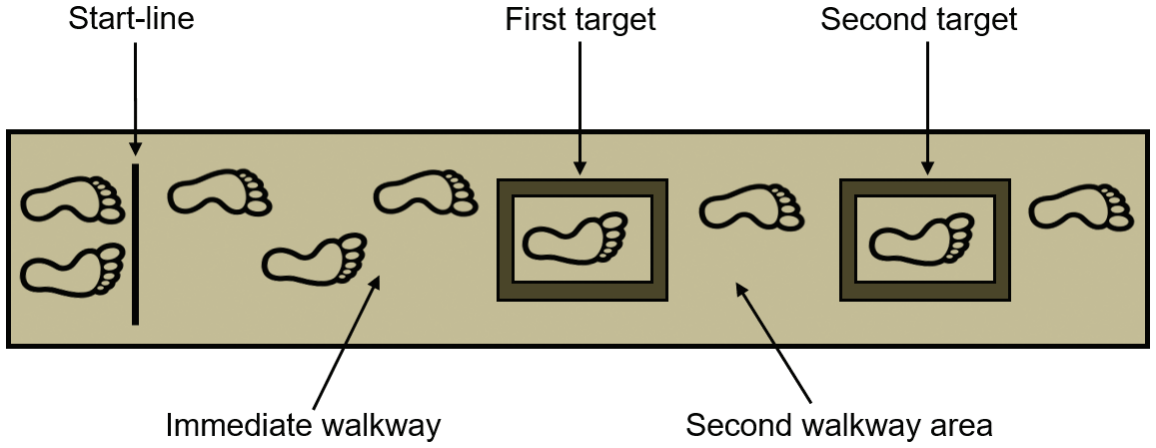
Schematic diagram of the walkway and precision stepping task. The foam targets had a border width and height of 4 cm (ie, the foam border was 4 cm wide and raised 4 cm from the walkway). The arrows denote the different areas of interest for which the walkway was separated for the gaze analysis.

The walking task and threat manipulation were identical to that used previously (Ellmers et al., 2020). The experimental task required participants to walk along a wooden path (width = 40 cm; length = 3.4 m) and step as accurately as possible into two rectangular foam targets, with whichever foot they wished (Figure 1). Each target was placed in one of the two possible locations (midpoint of the first target: either 1.5 m or 1.4 m from the walkway start-line; midpoint of the second target: either 2.5 m or 2.4 m from the start-line). Target locations were rearranged after every third trial to reduce familiarization—with the location of these targets randomized across participants. The foam targets had raised borders (foam border width and height = 4 cm), and the inside area of the target was 19 cm × 41.5 cm (width and length, respectively). Prior to the start of each trial, participants stood on the “start-line” (see Figure 1) with their eyes closed. This ensured that participants did not begin visually previewing the walkway prior to the start of the trial. Following an auditory “go” tone, participants opened their eyes and commenced the walking task.

Participants completed five trials under three separate conditions (15 trials total): (a) Baseline (completing the task at ground level); (b) Conscious movement processing (“CMP”; completing the task at ground level, but while directing conscious attention toward movement; see the section entitled “Conscious Movement Processing”); and (c) Threat (completing the task while the walkway was elevated 0.6 m above the laboratory floor). For each condition, participants completed five walks before moving onto the next condition. Conditions were presented in a counterbalanced order, and all trials were completed without a safety harness.

### Conscious Movement Processing

Heightened conscious movement processing was achieved by informing participants that they were required to direct conscious attention toward their movements, as they would be asked questions relating to their movement after certain trials (eg, “How many steps did you take between the two targets?” and “What foot did you begin walking with?”). Participants were informed that each question would only be asked once. Participants were presented with internal awareness questions after three randomly selected CMP trials, and the same questions were used for all participants. Participants were “informed” that any trials in which they answered incorrectly would be repeated, thus ensuring engagement with the manipulation. This method has been shown to successfully increase conscious movement processing to comparable levels as observed when people are anxious about falling (Ellmers & Young, 2018). Participants’ response accuracy was recorded as an additional manipulation check (see Results section).

In order to assess parity in the level of conscious movement processing between CMP and Threat conditions, participants were also asked internal awareness questions after three randomly selected Threat trials. We sought to ensure such between-condition parity to isolate behaviors casually associated with heightened conscious movement processing from those associated with other anxiety-related processes (as per Jackson, Ashford, & Norsworthy, 2006). It was thus reasoned that any comparable gaze behaviors observed between CMP (at ground level) and Threat are likely underpinned by a shared mechanism of heightened conscious movement processing.

### State Psychological Measures

To assess concern about falling (ie, fall-related anxiety), participants reported state levels of both balance confidence and fear of falling. Prior to each block of five walks, participants rated how confident they were that they could maintain balance and avoid a fall during the following condition (Zaback, Cleworth, Carpenter, & Adkin, 2015). Scores ranged from 0% (*not at all confident*) to 100% (*completely confident*). Participants completed this questionnaire while standing at the “start line” of the respective condition (ie, at ground level for Baseline and CMP; raised 0.6 m for Threat). After each block, participants rated their fear of falling (averaged across the previous five trials) on a scale ranging from 0% (*not at all fearful*) to 100% (*completely fearful*).

In an attempt to explore the attentional mechanisms which may underpin any differences in visual search behavior between CMP and Threat, a state measure of attentional allocation was collected after each block (Ellmers et al., 2020). This involved participants rating, on an 11-point Likert scale (1 = *never*, 11 = *always*), the degree to which they thought about or paid attention to the following sources of information during the previous five trials: Movement processes; Threats to balance; Worries or disturbing thoughts; Self-regulatory strategies; and Task-irrelevant information. The descriptions provided for each category can be found in Supplementary Table S1.

Participants also completed the Rating Scale of Mental Effort (RSME; Zijlstra, 1993) after each block of walks. This assessment required participants to rate the level of mental effort required to complete the previous five trials. The RSME was presented as a single continuum scale ranging from 0 to 150, with nine validated reference points along the scale (eg, “Absolutely No Effort,” “Some Effort,” “Extreme Effort,” etc.).

### Motor Performance

Participants completed all walks while fitted with reflective markers placed on the heel, mid-foot, and first metatarsal of both feet. Kinematic data were collected at 100 Hz using a Vicon motion capture system (Oxford Metrics, England) and passed through a low-pass Butterworth filter with a cutoff frequency of 5 Hz (Ellmers et al., 2020). The following motor performance variables were calculated (Ellmers et al., 2020): (a) Time to complete the walking trial (seconds between “go” tone and heel contact of final step on walkway); (b) Stance duration preceding the first and second target (time difference between heel contact and toe-off of foot initiating target step); and (c) stepping error (mm) in both the anterior-posterior (AP) and mediolateral (ML) planes for the first target and second target (ie, difference between co-ordinates of mid-foot marker and center of the target). Data were analyzed using custom algorithms in MATLAB version 7.11 (MathWorks, Natick, MA). Kinematic data were assigned a randomized code, to allow for blinded analysis, and variables were averaged across conditions.

### Gaze Behavior

Walks were completed while wearing a Mobile Eye-XG portable eye-tracking system (ASL, Bedford, MA). The eye-tracking system records participants’ gaze by contrasting the pupil and corneal reflection, allowing the superimposition of a point of gaze crosshair on a video of the environment recorded from a scene camera, which records wirelessly at 30 Hz. The eye-tracker was calibrated using a nine-point calibration protocol.

Visual fixations were defined as a gaze that endured on a single location (≤1° visual angle) for four frames or longer (Patla & Vickers, 1997). Fixation locations were classified as one of the four areas of interest (see Figure 1): (a) immediate walkway (the walkway prior to the first target); (b) the first target; (c) second walkway area (the walkway between the first and second target); and (d) the second target. These areas of interest were used to determine the duration spent fixating each location during the approach to the first target (until heel contact into the first target, calculated as the maximum vertical acceleration of the heel marker). Fixation duration data were normalized to individual trial length by presenting data as the percentage of time spent fixating each area of interest. As a further measure of visual previewing, the number of fixations made toward the second target (until heel contact into the first target) was also calculated. The location of the first fixation was also assessed. To determine this variable, each area of interest was allocated a number from 1 to 4 (immediate walkway = 1; first target = 2; second walkway area = 3; and second target = 4), with lower numbers indicating that the first fixation occurred in an area of interest closer to the walker’s feet.

Trials in which the point of gaze crosshair disappeared for the duration of four frames or more were discarded (Ellmers & Young, 2019). A total of 76 trials were analyzed for Baseline (*M* = 4.22 trials per participant), whereas 84 trials were analyzed for both CMP and Threat (*M* = 4.67 trials per participant, for each condition).

### Statistical Analysis

Separate repeated-measure ANOVAs were used to explore between-condition differences in all variables. We reported partial eta squared as measures of effect size. Bonferroni corrected post hoc tests were performed to follow-up any statistically significant results. Where data were non-normally distributed, separate Friedman tests were used instead. In these instances, any significant effects were followed up by separate Wilcoxon tests comparing each of the three conditions: Baseline, CMP, and Threat (Bonferroni corrected to 0.017). Due to the difficulties associated with calculating effect size for Friedman tests, effect sizes were calculated (and reported as *r* = *Z*/√*N*) instead for any Wilcoxon test follow-ups.

## Results

Summary statistics and measures of dispersion for each outcome measure are presented in Supplementary Table S2. Raw data are available at https://osf.io/ajhyq/.

### State Psychological Measures

#### Balance confidence

There was a significant main effect of Condition on balance confidence (*χ*^*2*^(2) = 20.47, *p* < .001). Post hoc tests revealed significantly lower balance confidence during Threat, when compared to both Baseline (*Z* = −3.31, *p* < .001, *r* = 0.78) and CMP (*Z* = −2.83, *p* = .003, *r* = 0.67). There was no significant difference between Baseline and CMP (*Z* = −1.87, *p* = .061, *r* = 0.44).

#### Fear of falling

There was also a significant main effect of Condition on fear of falling (*χ*^*2*^(2) = 12.00, *p* = .002). Post hoc tests revealed significantly greater fear of falling during Threat, when compared to both Baseline (*Z* = −2.23, *p* = .013, *r* = 0.55) and CMP (*Z* = −2.23, *p* = .013, *r* = 0.55). There was no significant difference between Baseline and CMP (*Z* = 0.00, *p* = 1.00, *r* = 0.00).

#### Attentional focus

There was a significant main effect of Condition on the amount of attention directed toward movement processes (*χ*^2^(2) = 14.00, *p* = .001). Compared to Baseline, participants reported significantly greater attention toward movement processes during both CMP (*Z* = −2.52, *p* = .006, *r* = 0.59) and Threat (*Z* = −3.12, *p* = .001, *r* = 0.74). Participants reported statistically comparable levels of conscious movement processing during CMP and Threat (*Z* = −0.05, *p* = .96, *r* = 0.01). There was a significant main effect of Condition on the amount of attention directed toward self-regulatory processes (*χ*^2^(2) = 10.87, *p* = .004). During Threat, participants directed significantly greater attention toward self-regulatory processes compared to both Baseline (*Z* = −2.67, *p* = .004, *r* = 0.63) and CMP (*Z* = −2.39, *p* = .009, *r* = 0.56). Participants reported statistically comparable levels of self-regulatory strategies during Baseline and CMP (*Z* = −0.71, *p* = .48, *r* = 0.17). There was also a significant main effect of Condition on the amount of attention directed toward worries/disturbing thoughts (*χ*^2^(2) = 6.50, *p* = .039). However, subsequent post hoc tests revealed no significant differences between any conditions (*p*s > .033, *r*s > 0.44). There was no significant main effect of Condition on the amount of attention directed toward either threats to balance (*χ*^2^(2) = 0.74, *p* = .69) or task-irrelevant information (*χ*^2^(2) = 1.08, *p* = .58).

#### Internal awareness response accuracy

Mean response accuracy for internal awareness questions was identical in both CMP and Threat (*Median* = 3 out of 3, *Interquartile Range* = 1.00, *Z* = 0.00, *p* = 1.00); with five participants providing a single incorrect answer during CMP and five other participants answering a single question incorrectly during Threat.

#### Mental effort

There was a main effect of Condition on mental effort (*χ*^*2*^(2) = 16.57, *p* < .001). Compared to Baseline, participants reported significantly greater mental effort during both CMP (*Z* = −2.68, *p* = .004, *r* = 0.63) and Threat (*Z* = −3.27, *p* < .001, *r* = 0.77). Participants reported statistically comparable levels of mental effort between CMP and Threat (*t*(17) = −1.53, *p* = .14, *d* = 0.19).

### Motor Performance Measures

#### Time to complete the walking task

There was a significant main effect of Condition on task completion times (*F*(1.37, 23.35) = 15.09, *p* < .001, *ƞp*^2^ = 0.47). Compared to Baseline, participants took significantly longer to complete the task during both CMP (*p* = .015) and Threat (*p* = .001). Completion times were also significantly longer during Threat, than during CMP (*p* = .020).

#### Stance times

**Figure 2.**
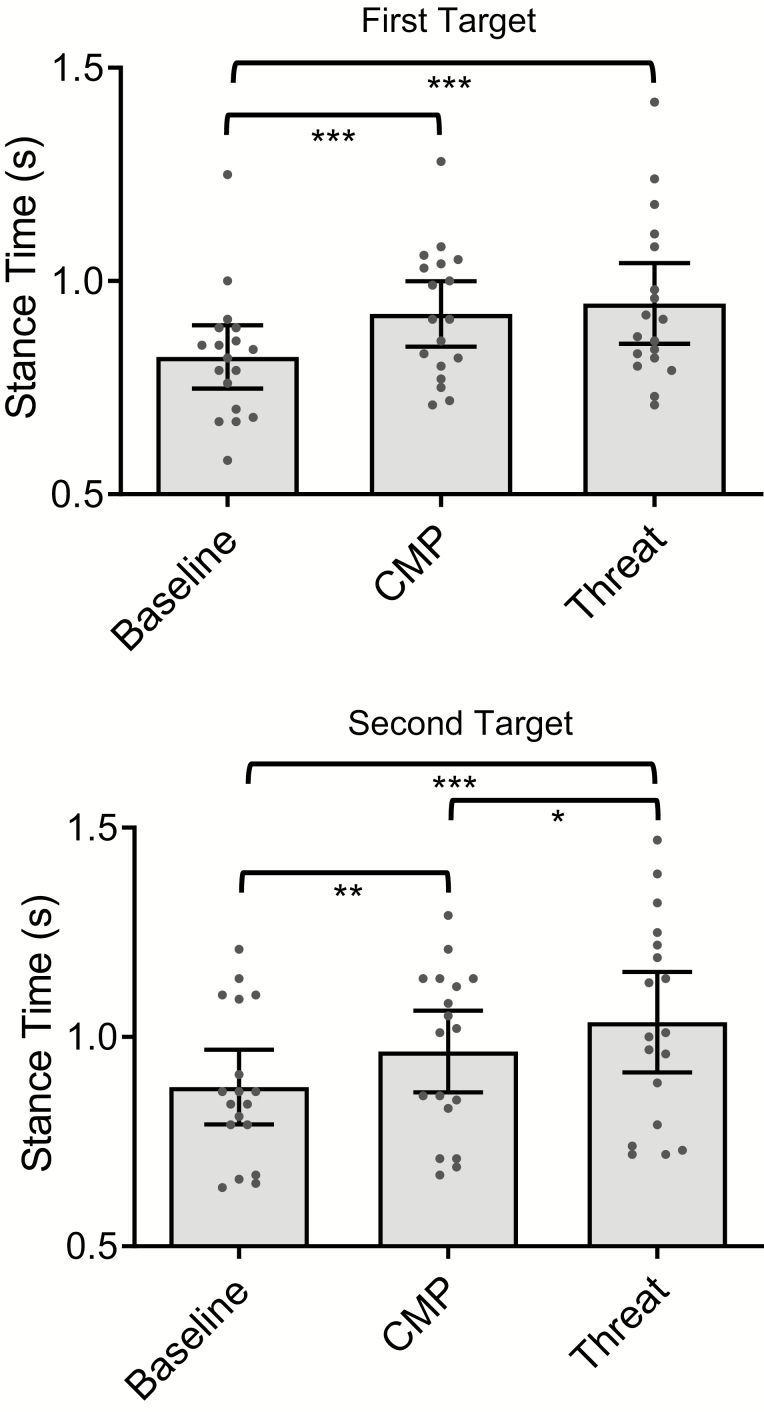
Stance durations (s) preceding the first target (top) and second target (bottom), during conditions of Baseline, CMP, and Threat, *p < .05, **p < .01, ***p < .001 (mean ± 95% confidence intervals and individual data points).

There was a significant main effect of Condition on stance durations preceding the first target (*F*(2, 34) = 16.84, *p* < .001, *ƞp*^2^ = 0.50), with significantly longer stance durations during both CMP (*p* < .001) and Threat (*p* < .001), compared to Baseline; but comparable stance durations between CMP and Threat (*p* = .88). These data are presented in Figure 2A. There was also a significant main effect of Condition on stance durations preceding the second target (*F*(2, 34) = 21.95, *p* < .001, *ƞp*^2^ = 0.56), with significantly longer stance durations during both CMP (*p* = .002) and Threat (*p* < .001), compared to Baseline. Stance durations were also significantly longer during Threat, compared to CMP (*p* = .022). These data are presented in Figure 2B.

#### Stepping error

There was a significant main effect of Condition on AP stepping errors into the first target (*F*(2, 34) = 2.53, *p* = .040, *ƞp*^2^ = 0.17). However, subsequent post hoc tests revealed no significant differences between any conditions (all *p*s > 0.069). There was also a significant main effect of Condition on ML stepping errors for the first target (*χ*^*2*^(2) = 7.11, *p* = .029). However, post hoc

 follow-up tests revealed no significant differences between any conditions (*p*s > 0.053, *r*s > 0.42). Regarding the second target, there was a lack of significant main effect for stepping errors in either AP (*F*(2, 34) = 0.22, *p* = .81, *ƞp*^2^ = 0.01) or ML directions (*χ*^*2*^(2) = 3.44, *p* = .18).

### Gaze Behavior Measures

#### First fixation location

There was a main effect of Condition on the location of participants’ first fixation (*χ*^2^(2) = 19.32, *p* < .001). Compared to Baseline, participants’ first fixation occurred significantly closer to the walkway start during both CMP (*t*(17) = 2.68, *p* = .016, *d* = 0.48) and Threat (*Z* = −3.46, *p* < .001, *r* = 0.82). Participants’ first fixations were also located significantly closer to the walkway start during Threat, compared to CMP (*Z* = −2.50, *p* = .007, *r* = 0.59).

#### Fixation durations

**Figure 3.**
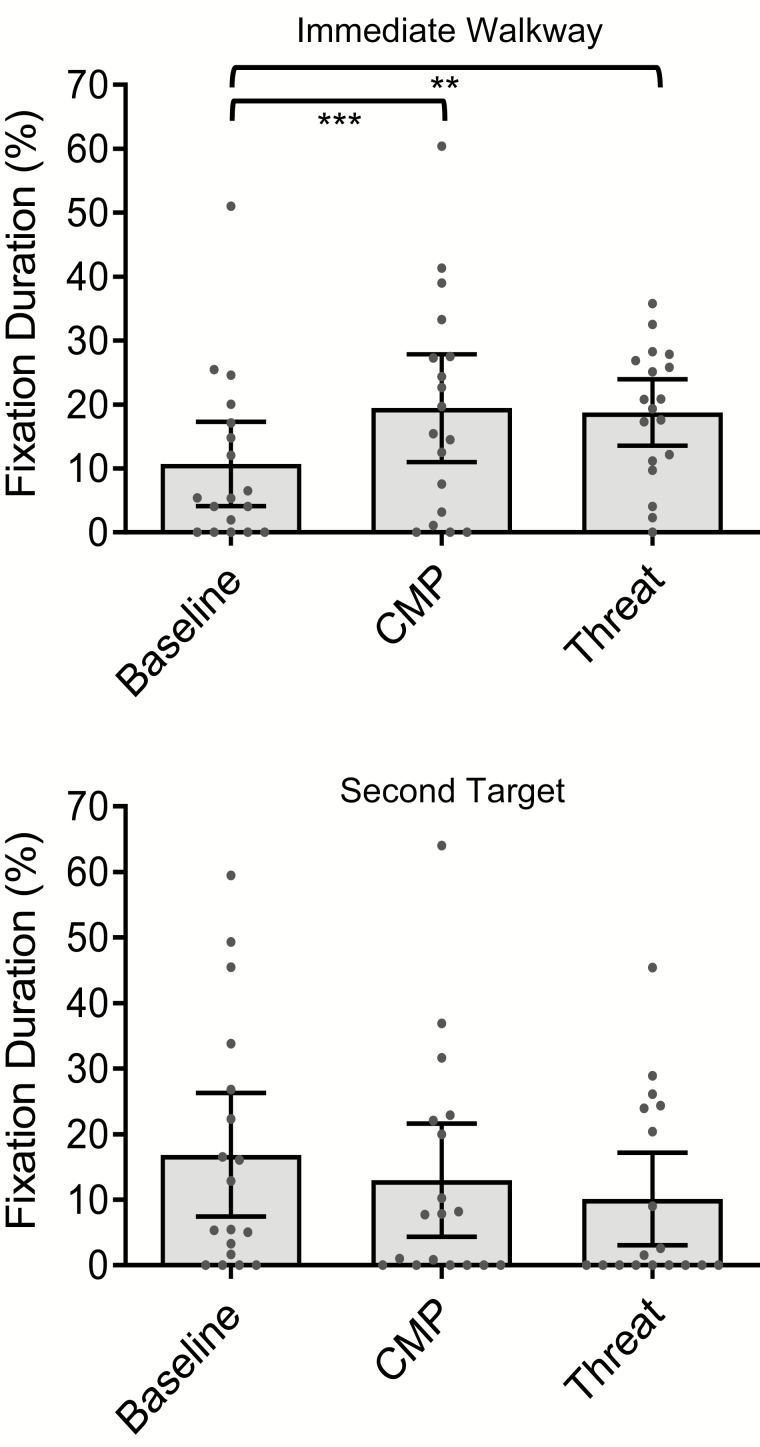
The percentage of time spent fixating the immediate walkway area preceding the first target (top) and the second target (bottom) during Baseline, CMP, and Threat conditions, **p < .01, ***p < .001 (mean ± 95% confidence intervals and individual data points).

There was a main effect of Condition on the percentage of time spent fixating the immediate walkway (*χ*^2^(2) = 12.80, *p* = .002). Compared to Baseline, participants spent a significantly greater percentage of time fixating the immediate walkway during both CMP (*Z* = −3.21, *p* < .001, *r* = 0.76) and Threat (*Z* = −2.85, *p* = .002, *r* = 0.67). The percentage of time spent fixating the immediate walkway was statistically comparable between CMP and Threat (*t*(17) = 0.24, *p* = .81, *d* = 0.05). These data are illustrated in Figure 3A. There was a lack of significant main effect of Condition on the percentage of time spent fixating either the first target (*F*(2,34) = 0.34, *p* = .71, *ƞp*^2^ = 0.02) or the second walkway area (*χ*^2^(2) = 3.13, *p* = 0.21). While Figure 3B illustrates an overall trend for participants to reduce the time spent fixating the second target during CMP and Threat, the main effect of Condition on the overall percentage of time spent fixating the second target did not reach statistical significance (*χ*^2^(2) = 4.97, *p* = .08).

#### Visual previewing of future stepping constraints

**Figure 4.**
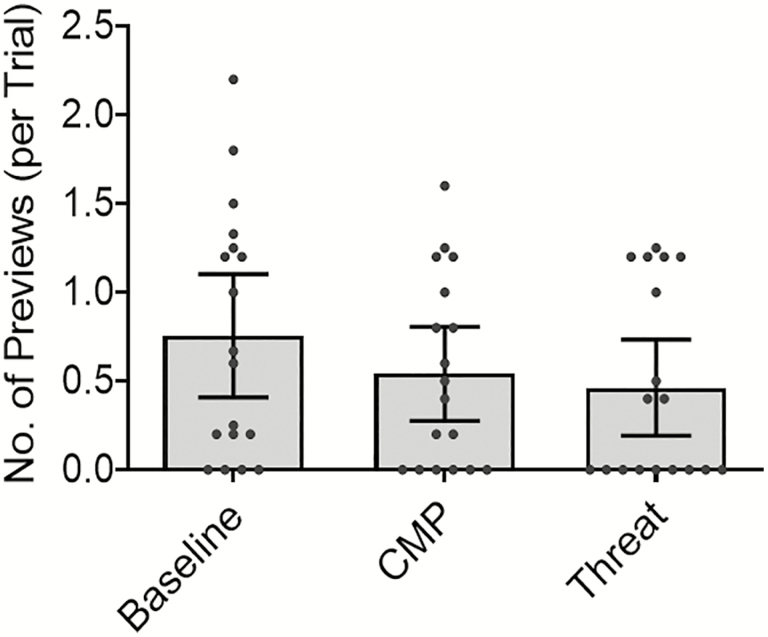
The average number of previewing fixations (per trial) made toward the second target (when approaching the first target), during conditions of Baseline, CMP, and Threat (mean ± 95% confidence intervals and individual data points).

While Figure 4 illustrates an overall trend for participants to reduce the number of previewing fixations made toward the second target during CMP and Threat, the main effect of Condition on the overall number of previewing fixations was nonsignificant (*χ*^*2*^(2) = 4.19, *p* = .12).

## Discussion

The present study investigated the influence of conscious movement processing and fall-related anxiety on older adults’ visuomotor control during locomotion. Specifically, we explored the extent to which visual search patterns observed in older adults anxious about falling (Ellmers et al., 2020; Young et al., 2012)—behaviors likely to reduce safety during locomotion (Young & Williams, 2015)—might be a consequence of attempts to consciously process walking movements. Given the identical mean levels of self-reported state movement processing observed between CMP and Threat (and identical response accuracy to internal awareness questions), the experiment successfully induced similar levels of conscious movement processing between these conditions.

As predicted, we observed largely comparable gaze behaviors between CMP and Threat. Previous research has reported that older adults will display markedly altered visual search behavior when anxious about falling (Ellmers et al., 2020; Young et al., 2012). Specifically, older adults who are anxious will direct preferential attention toward proximal areas of their walking path, at the expense of previewing future environmental constraints. These behaviors have been suggested to represent a prioritization of the visual information needed to consciously control/monitor each individual step—at the expense of planning future stepping actions (Ellmers & Young, 2019). Our results provide strong support for such interpretation.

During both experimental conditions, participants appeared to visually prioritize proximal walkway areas (1–2 steps ahead) to a greater extent than during Baseline. As illustrated in Figures 3B and 4, older adults who consciously process their walking movements will visually prioritize proximal (eg, immediate walkway), over distal (eg, second or first target), areas of the walking path. These findings are in general agreement with work conducted during static postural control tasks which have reported associations between conscious movement processing and the direction of attention toward more proximal environmental cues (Wulf, 2013). Our findings are also in line with previous research reporting significant associations between self-reported state conscious movement processing and gaze behaviors indicative of heightened on-line (and reduced feedforward) visual control of locomotion in older adults—even when controlling for age, physical and cognitive functioning (Ellmers et al., 2020). Taken together, we propose that these findings suggest that the changes in visual search observed when anxious about falling are, at least in part, a consequence of heightened conscious movement processing.

The singular significant difference in visual search observed between CMP and Threat related to the location of the first fixation. Participants’ first fixation occurred significantly closer to the start of the walkway during Threat. Staab (2014) proposed that anxious individuals exhibit an attentional bias for external threat-related stimuli during locomotion. We thus interpret these more proximal first fixations to represent an initial hypervigilance toward immediate threats to balance (ie, the immediate walkway when walking on the elevated walkway in the present study; or, for example, the immediate ground if walking on an uneven surface in a real-world setting). Interestingly, when asked, participants in the present research did not report directing greater attention toward external threats to balance during Threat. Hence, such hypervigilance may represent a largely subconscious behavioral response.

The adoption of hypervigilant behavior during Threat is in line with predictions of Attentional Control Theory (ACT; Eysenck, Derakshan, Santos, & Calvo, 2007), which suggests that anxiety increases the influence of the stimulus-driven attentional system (eg, immediate/salient threats) at the expense of the goal-directed system (eg, proactively scanning one’s whole environment and planning future stepping actions). As such, we propose that the initial hypervigilance observed during conditions of Threat likely represents preferential attention allocated toward detecting the source of threat, with subsequent conscious on-line (visual) control selected as the behavioral response to mitigate this perceived threat and avoid a fall occurring. Such conscious movement processing appeared to constitute an increase in both short-term planning (eg, planning individual steps rather than planning future adaptive stepping movements) and on-line control (eg, guiding the trajectory of the step itself).

It is worth noting that another study has recently reported a lack of association between conscious movement processing and visual search during adaptive locomotion in older adults (Uiga et al., 2020). However, this study restricted analyses to self-reported levels of generalized trait conscious movement processing (ie, the degree to which an individual consciously controls/monitors movement in general, not necessarily during gait). Recent findings suggest that levels of self-reported trait conscious movement processing may not reliably translate to the context of gait (Young, Ellmers, Kinrade, Cossar, & Cocks, Under Review). It is also noteworthy that the “distal” stepping constraint used by Uiga et al. (2020) consisted of a narrow gap between two obstacles (unlike the precision stepping task used here). Discrepancies between our respective findings could therefore be due to differences in either the measurement used to assess conscious movement processing, and/or the task constraints and associated need for participants to proactively fixate and plan future stepping actions.

In addition to the aforementioned significant between-condition differences in initial fixation locations, participants also displayed significantly different walking behaviors between CMP and Threat. Specifically, participants took longer to complete the walking task during Threat, in addition to displaying significantly longer stance durations preceding the second target. We propose that, given the increased negative consequences associated with a misplaced step while walking on the elevated walkway, these slower adaptive stepping movements represent a compensatory (and likely consciously processed) response to ensure that these actions are correctly programmed and executed.

While self-reported levels of conscious movement processing were equivalent between CMP and Threat, as were the large majority of eye-tracking variables, it is possible that the nature of conscious movement processing may have been qualitatively different between experimental conditions. Previous research has reported increased internal awareness in older adults during conditions of postural threat (as indicated by increased accuracy on comparable internal awareness questions; Young et al., 2016). Such awareness (and any manipulation used to induce this form of conscious processing) relates, however, primarily to movement *monitoring*, rather than *control.* We decided against using “conscious control” instructions in the present research, as we deemed that such manipulations, whereby individuals are instructed to focus on controlling a certain aspect of their movement (Mak et al., 2020), are unlikely to capture the complex, multifaceted nature of the conscious movement strategies that anxious older adults would spontaneously adopt when anxious (eg, Ellmers et al., 2019). Another issue, however, relates to our “internal awareness” questions likely drawing attention—and associated visual fixations—toward participants’ footfall positions. Previous cross-sectional research has reported that higher conscious movement processing is associated with more accurate answers to comparable internal awareness questions (Uiga et al., 2015; Young et al., 2016) and that self-reported conscious movement processing is associated with gaze behaviors comparable to that observed here (Ellmers et al., 2020). Therefore, we suggest that the alterations in gaze behavior observed during our CMP manipulation would also be observed during “spontaneous” conscious movement processing, such as when anxious about falling.

For the highly functioning older adults in our study, the visual search patterns associated with conscious movement processing did not appear to directly reduce safety (given the lack of increased stepping errors observed from Baseline). Our data do, however, suggest an indirect impact on safety, as conscious processing was found to impair attentional processing efficiency (ie, reported an increase in the level of mental effort required to complete the task during CMP) and reduced movement efficiency (ie, increased stance durations preceding the first and second target during CMP and Threat). These findings indicate that conscious movement strategies are both cognitively demanding and less efficient. As such, we suggest that consciously processing walking movements will likely reduce safety during complex locomotor tasks, such as those requiring rapid stepping movements, or during scenarios where attentional capacity is taxed. Indeed, previous research has shown significantly greater stepping errors in young adults consciously processing their stepping movements while performing a cognitively demanding simultaneous task (Ellmers & Young, 2018).

This work has several limitations that need to be discussed. Firstly, the participants were all healthy, high-functioning older adults at a low risk of falling. Older adults deemed to be at a high risk of falling will typically consciously process their walking movements during baseline/control conditions (Ellmers et al., 2020). We thus reasoned that isolating specific behaviors related to conscious movement processing would be difficult in this population. While the current task (and its predecessors; Chapman & Hollands, 2006b; Ellmers et al., 2020; Uiga et al., 2020; Young et al., 2012), challenges functional processes of feedforward and online control of adaptive gait, its ecological validity is limited. Therefore, future work should seek to replicate these findings in a task more generalizable to naturalistic settings.

## Conclusion

This study supports the assertion that conscious movement processing is, at least partially, responsible for the (suboptimal) visual search patterns typically observed in older adults anxious about falling. While the current data cannot be considered direct evidence of an underlying shared mechanism, our results indicate that consciously processing walking movements results in greater time spent fixating immediate walkway areas (1–2 steps ahead) at the expense of feedforward visual planning; behaviors associated with reduced stepping safety in older adults deemed to be at a high risk of falling (Ellmers et al., 2020). While indiscriminately restricting *all* conscious attempts to process posture and gait will likely have a detrimental effect (Boisgontier et al., 2013), future research should explore whether reducing excessive (or unwarranted) conscious processing can enhance visuomotor control and stepping performance during adaptive gait in older adults deemed to be at a high risk of falling.

## Conflict of Interest

The authors declare that they have no competing interests.

## Supplementary Material

gbaa081_suppl_Supplementary_Data

## Footnotes

^1^ However, the data reported herein are new and not a reanalyzed subset of data from this previous work.

^2^ Note, these participants completed tests of visual acuity and contrast sensitivity while wearing correcting lenses.
