# Supplementary material for: Conscious Movement Processing, Fall-Related Anxiety, and the Visuomotor Control of Locomotion in Older Adults
Source: J Gerontol B Psychol Sci Soc Sci. 2020 Aug 6;75(9):1911–20. doi: 10.1093/geronb/gbaa081 (PMC7566972; doi:10.1093/geronb/gbaa081)
Supplement: gbaa081_suppl_Supplementary_Data [file gbaa081_suppl_supplementary_data.docx]

**Supplementary Table 1.** The descriptions provided to participants for each category in the state attentional measure administered.

| **Category** | **Description** |
| --- | --- |
| Movement processes | *Attempts to consciously control or monitor movement, e.g., focusing on picking up your feet, or controlling either step length or walking speed* |
| Threats to balance | *Focusing on anything in the environment which may cause you to trip up or lose your balance* |
| Worries or disturbing thoughts | *E.g., Thoughts relating to falling and the potential negative consequences of this* |
| Self-regulatory strategies | *Any coping strategy that helps you feel, think and behave in the way you want, e.g., focusing on controlling your breathing or telling yourself “you can do it!”* |
| Task-irrelevant information | *Thoughts unrelated to the walking task, e.g., thinking about what you are having for dinner or letting one’s mind wander* |

**Supplementary Table 2.** Mean, standard error of the mean (*SEM*) and main-effect *p* values for comparisons between Baseline, CMP and Threat trials for all outcome variables.

|  | **Baseline** | | **CMP** | | **Threat** | |  |
| --- | --- | --- | --- | --- | --- | --- | --- |
|  | Mean | *SEM* | Mean | *SEM* | Mean | *SEM* | *p* |
| Balance confidence (%) | 92.61 | 3.03 | 87.50 | 4.68 | 77.22^†Ꞝ^ | 4.97 | <.001 |
| Fear of falling (%) | 1.48 | 0.81 | 1.11 | 0.76 | 9.44^†Ꞝ^ | 3.66 | .002 |
| Mental effort (0-150) | 18.89 | 4.65 | 31.94^†^ | 5.24 | 36.11^†^ | 5.14 | <.001 |
| *Attention directed towards…* |  |  |  |  |  |  |  |
| …Movement processes (1-11) | 4.22 | 0.74 | 7.00^†^ | 0.81 | 7.00^†^ | 0.69 | .001 |
| …Threats to balance (1-11) | 1.61 | 0.45 | 1.28 | 0.18 | 1.89 | 0.49 | .692 |
| …Worries or disturbing thoughts (1-11) | 1.11 | 0.11 | 1.06 | 0.06 | 1.72 | 0.42 | .039 |
| …Self-regulatory strategies (1-11) | 2.06 | 0.42 | 2.22 | 0.36 | 3.17^†Ꞝ^ | 0.47 | .004 |
| …Task-irrelevant information (1-11) | 1.56 | 0.44 | 1.11 | 0.11 | 1.06 | 0.06 | .584 |
| Time to complete the task (s) | 5.28 | 0.22 | 5.70^†^ | 0.23 | 6.51^†Ꞝ^ | 0.39 | <.001 |
| Stance duration (s), first target | 0.82 | 0.04 | 0.92^†^ | 0.04 | 0.95^†^ | 0.05 | <.001 |
| Stance duration (s), second target | 0.88 | 0.04 | 0.97^†^ | 0.05 | 1.04^†Ꞝ^ | 0.06 | <.001 |
| First target, AP stepping error (mm) | 29.53 | 3.23 | 29.61 | 3.48 | 24.41 | 2.63 | .040 |
| First target, ML stepping error (mm) | 14.00 | 1.93 | 16.22 | 1.67 | 18.69 | 1.65 | .029 |
| Second target, AP stepping error (mm) | 33.96 | 4.41 | 36.10 | 4.36 | 35.23 | 3.42 | .805 |
| Second target, ML stepping error (mm) | 14.47 | 1.61 | 17.78 | 2.04 | 16.45 | 1.80 | .179 |
| First fixation location (1-4)  (1) Immediate walkway  (2) First target  (3) Second walkway  (4) Second target | 2.10^a^ | 1.16^b^ | 1.90^a†^ | 1.10^b^ | 1.60^a†Ꞝ^ | 0.65^b^ | <.001 |
| Immediate walkway fixation duration (%) | 10.69 | 3.13 | 19.43^†^ | 3.98 | 18.75^†^ | 2.45 | .002 |
| First target fixation duration (%) | 61.35 | 5.39 | 59.11 | 4.31 | 58.90 | 4.31 | .714 |
| Second walkway fixation duration (%) | 11.11 | 2.47 | 8.49 | 1.91 | 12.22 | 2.29 | .209 |
| Second target fixation duration (%) | 16.86 | 4.46 | 12.97 | 4.10 | 10.13 | 3.34 | .084 |
| No. previewing fixations (avg. per trial) | 0.76 | 0.16 | 0.54 | 0.13 | 0.46 | 0.13 | .123 |

Note: ^a^ = median (rather than mean), ^b^ = interquartile range (rather than *SEM*), ^†^ = statistically different from Baseline, ^Ꞝ^ = statistically different from CMP
